# Stakeholder engagement in the health policy process in a low income country: a qualitative study of stakeholder perceptions of the challenges to effective inclusion in Malawi

**DOI:** 10.1186/s12913-021-07016-9

**Published:** 2021-09-18

**Authors:** Sarah C. Masefield, Alan Msosa, Florence Kasende Chinguwo, Jean Grugel

**Affiliations:** 1 Interdisciplinary Global Development Centre, University of York, York, YO10 5DD UK; 2 Health Economics and Policy Unit, Department of Health Systems and Policy, School of Public Health and Family Medicine, College of Medicine, University of Malawi, Blantyre, Malawi; 3 Department of Politics, University of York, York, YO10 5DD UK

**Keywords:** Stakeholder engagement, Malawi, SDG 3, Civil society organisations, Health policy

## Abstract

**Background:**

Inclusive engagement in healthcare policies and decision-making is essential to address the needs of patients and communities, reduce health inequities and increase the accountability of the government. In low income countries such as Malawi, with significant health challenges, stakeholder inclusion is particularly important to improve performance and service delivery. The 2017 National Health Plan II (NHP II) and accompanying Health Sector Strategic Plan II (HSSP II) aimed to improve the functioning of the healthcare system. The Ministry of Health for Malawi intended to involve all key health sector stakeholders in their development. This study explores the extent of stakeholder engagement in the health policy process through local level stakeholders’ perceptions of their involvement in the NHP II and HSSP II.

**Methods:**

A qualitative study design was used. Interviews were conducted with 19 representatives of organisations operating at the local level, such as CSOs and local government. Open questions were asked about experiences and perceptions of the development of the NHP II and HSSP II. Inductive content analysis was performed.

**Results:**

Stakeholders perceived barriers to inclusive and meaningful engagement in the health policy process. Five categories were identified: tokenistic involvement; stakeholder hierarchy; mutual distrust; preferred stakeholders; no culture of engagement.

**Conclusions:**

Serious challenges to the meaningful and equitable engagement of local level stakeholder groups in the health policy process were identified. Issues of trust, accountability and hierarchy in donor-citizen-government relations must be addressed to support stakeholder engagement. Engagement must go beyond tokenism to embed a range of stakeholders in the process with feedback mechanisms to ensure impact from their contributions. Local level stakeholders can be empowered to advocate for and participate in consultation exercises alongside greater top-down efforts to engage stakeholders via diverse and inclusive methods. These issues are not unique to Malawi or to health policy-making.

## Background

Stakeholder engagement throughout the policy development cycle (problem identification, agenda setting, policy formulation, adoption, implementation, evaluation) is essential for understanding the needs of different groups and communities, such as civil society organisations and donors who may have different priorities, and for increasing equity in policy [1–3]. It can give additional legitimacy by providing evidence to support and shape policies and increase accountability of the government to stakeholders thus achieving greater policy implementation [4–6]. National health policies and plans with comprehensive stakeholder engagement throughout the policy cycle tend to be more robust and have more effective implementation [2, 7]. As such stakeholder engagement is considered an essential part of democracy in both developed and developing countries [5].

The taxonomy of the 7Ps of Stakeholder Engagement can be used to define the key health stakeholder groups: patients and the public, providers, purchasers, payers, policy makers, product makers and principal investigators [8]. Also useful conceptually is Alemanno’s three components of effective stakeholder engagement: 1) public communication; 2) public consultation; and 3) public participation [5]. The World Health Organization specifies that consultation should facilitate an inclusive dialogue which aims to build consensus on current provision and on the values, goals and overall policy directions that will guide health policy [9]. However, the extent, commitment and capacity to deliver effective stakeholder engagement, including of marginalised groups, varies considerably between countries and health policy areas [5]. The engagement process is shaped by political will, established cultures of stakeholder engagement, hierarchical social relationships, lobbying by some stakeholders (but not others) for inclusion, poor understanding by stakeholders of policy processes and institutions (policy literacy), and limited resources, meaning that equitable and inclusive consultation is often difficult to achieve [5, 10].

Malawi is a democratic country with a constitution which promotes fundamental liberties and freedoms as well as a set of institutions to promote, protect and safeguard democratic governance [11]. Unlike in the one-party era, in principle, policy-making is no longer an exclusive preserve of the president: the executive (the cabinet and civil servants) initiates policies and the legislature is involved in debating and approving bills [12, 13]. The move to democratic government brought in the need to consult with diverse stakeholders in the policy formulation process to, as stated in section seven of the constitution, ‘embody the express wishes of the people of Malawi’ [11, 14].

In 2017, the Government of the Republic of Malawi Ministry of Health produced the National Health Policy II (NHP II), which is closely aligned with the Sustainable Development Goal (SDG) 3 to ensure healthy lives and wellbeing for all at all ages [15]. The MoH states that it ‘initiated a bottom up approach, highly participatory, and multi-stakeholder consultations to develop the policy’ which aims to ‘properly guide stakeholders in the implementation of initiatives to improve the functioning of the health system’ [15]. It identifies stakeholders who have provided ‘technical contributions towards the development of the policy’ (participants from districts and central hospitals, MoH Departments, regulatory bodies, the Parliamentary Committee on Health, donor and implementing partners) and stakeholders who are responsible for the implementation of the policy’s seven priority areas, including civil society organisations (CSOs). Patients and advocacy groups are highlighted as key stakeholders but no further reference to their involvement in the policy process is made.

An accompanying strategic framework, the Health Sector Strategic Plan II (HSSP II), outlines the objectives, strategies and activities and guiding resources for the period 2017–2022 [16]. The document states that ‘all key stakeholders in the health sector’ were included in the steering committee to guide and coordinate the development of the framework [16]. Stakeholders were invited to consultative workshops, technical working groups and visits to institutions, departments and programmes with the MoH and other Ministries, Departments and Agencies, District Health Offices (DHOs), Central Hospitals, health regulatory bodies, the private sector, donors and CSOs [16]. Walsh et al. performed a stakeholder analysis of the actors that influenced the development of the HSSP II, interviewing members of the MoH and donors. They reported the involvement of (unnamed) CSOs, non-government organisations (NGOs) and District Health Officers but these stakeholders were not interviewed. Whilst having a seat at the table is essential for stakeholder engagement, it does not mean that their views informed decision-making. Due to decentralisation, local government (district and city level) is not part of central government or the referral health system; therefore, like other non-government stakeholders such as CSOs and NGOs, they have a role advocating for the health needs of their populations in the development and implementation of national health policies.

There is widespread global recognition that local level organisations are often the stakeholders with the greatest knowledge of the current health situation and unmet health needs, and of course they will be directly affected by any regulation [5]. Our aim, then, was to reflect upon the effectiveness of stakeholder engagement in health policy development in Malawi by exploring the perceptions of local level government and organisations as to their engagement in the development of health policy in Malawi since 2017, chiefly the National Health Policy II and accompanying Health Strategic Plan II and through this process to contribute to wider discussions about stakeholder engagement in health policy-making in low and middle income countries (LMICs). In our study, we draw on Alemanno’s three components, identifying effective stakeholder engagement in health policy-making as opportunities provided by central government for communication, consultation and participation that local level stakeholders consider accessible and timely, through which they can exert influence.

## Methods

This study arises from our work on challenges to effective governance of the Malawian healthcare system, where we first identified a potential gap between being consulted and influencing policy decision-making for these stakeholder groups [17]. The interview data used in this study derives from a subset of individuals (*n* = 19/22) and questions from wider-ranging semi-structured interviews on health stakeholder perceptions of their involvement in the health system and healthcare decision-making in Malawi, including effective governance and stakeholder engagement in policy-making. Interviews with three local-government representatives were excluded from this study as not containing content relating to stakeholder engagement. The study methods (and limitations) are described extensively elsewhere [17]; therefore, we present only a brief description here, with a focus on the areas where the methods differ.

For the wider research project which encompasses this study, ethical approval was received from both the University of York (6 July 2018) and from the College of Medicine in the University of Malawi (16 October 2018). The interviews were conducted by AM between December 2018 and February 2019 in a combination of English and Chichewa.

### Participants

We used a purposive sampling strategy following a mapping exercise to identify health stakeholders working in decision-making roles at the local or service level of the government-funded health system or who have advocated for change in the health sector. This exercise was performed by AM and Thanzi la Onse project partners in the College of Medicine at the University of Malawi and the Oversees Development Institute who have local and specialist knowledge from conducting health research in Malawi. Full details of the mapping exercise and sampling strategy are described in our related publication [17]. Verbatim transcripts from face-to-face interviews with 19 individuals identified via the mapping exercise form the empirical basis of this paper They are representatives from: NGOs (*n* = 5); CSOs (*n* = 9); local government (*n* = 3) and government-funded but otherwise independent institutions (e.g. hospitals and commissions on specific disease areas; *n* = 2).

### Data collection

The subset of interview questions which provided the data for this study were: 1) do you collaborate with the government by influencing health policy and/or budgeting? If yes, to what extent were you and your organisation involved in the development of the national health policy and health sector strategic plan and or the health budget; and 2) do you think there is an opportunity for you at your level to get involved in health policy-making?

Due to the potentially sensitive nature of the wider interview content, and the linkage between this and another study [17], every effort has been made to anonymise the individual participants in the reporting of this research. Attribution is made by type of organisation and participant number only (the numbers differ from those used in the linked publication to further prevent participant identification).

### Data analysis

The analysis was performed by SM using inductive thematic analysis [18]. This meant that instead of producing a coding framework prior to commencing the analysis, we used an iterative process of identifying and refining themes within and between transcripts as we moved through analysis of the separate transcripts and field notes. All transcripts were reviewed after the initial analysis stage to ensure all relevant units of data (words, sentences and paragraphs) had been identified and combined/grouped with similar content to form the themes [18]. While this process was not highly structured, it was comprehensive and exploratory, allowing themes to emerge directly from the data. The emergent themes with a sample of illustrative quotes were discussed with JG who helped conceive the study and AM who performed the interviews and produced the transcripts. This procedure and review of the manuscript by our local partner FK, helped to reduce any potential bias.

We did not seek during the interviews or the analysis to identify the extent to which different interviewees or stakeholder groups agreed on specific issues. For this reason, a data saturation approach was not used in the analysis. Instead, all relevant units of data are presented: where available, direct quotations are used to illustrate interviewee perceptions; elsewhere, impressions and scenarios described by the interviewees and recorded in the field notes are presented. We also note agreement or disagreement between interviewee perceptions and indicate how many interviewees expressed the same opinion.

The analysis was performed between November 2019 and January 2020 in NVivo 12 software.

## Results

As health stakeholders, every interviewee expressed a desire to be heard and to use their knowledge to influence central government health policy-making, ‘as an organisation that also deals with health issues, there ‘should have been some form of involvement for us to input into the documents’ (P11, CSO). They felt that being involved in policy decision-making would communicate the policy needs of their members and the communities they serve, helping hold those responsible for policy implementation to account:

‘[Name of organisation] is the mouthpiece of what happens on the ground regarding HIV and AIDS in Malawi. They are the organisation that can tell the nation and stakeholders what happens in Malawian communities […] There is need for CSOs to position themselves as key stakeholders in mainstream health so that they are included in such policy and strategy [HSSP II and NHP II] processes. By getting involved at such policy or strategy level, there is an opportunity for the organisations to influence donor priorities, demand services or accountability’ (P10, CSO).

They also saw it as their role to provide some social context to the scientific discussions that they viewed as often dominating the policy development process:

‘the focus of the MoH was too biomedical, and often overlooked the social or political elements of health. By embedding the engagement of CSOs and NGOs throughout the policy development process, the wider determinants of health would be considered more routinely as these organisations often prioritise addressing social issues in their own work’ (P10, CSO).

Of the 19 interviewees, 11 reported having some involvement in the development or validation of the HSSP II, and only one in the development of the NHP II. Yet, most of the interviewees described feeling powerless to influence the content of health policy in Malawi, because they were either not consulted at all, not engaged throughout the policy life cycle (i.e. they were only consulted at the implementation not the development stages), insufficient time was allocated for stakeholder consultation, or their contributions were disregarded (P17, P11, P12, P10, P7, P15, P19). There was a perceived hierarchy of stakeholder engagement, with the citizens at the bottom having no influence and donors at the top with the greatest leverage over the government because of their substantial financial contribution to healthcare in Malawi. The interviewees described some methods they had used to help foster a more widespread culture of stakeholder engagement, starting with their own organisations’ structures, activities, and partnerships.

Five key challenges to effective stakeholder engagement in health policy (the themes) emerged from the data: tokenistic involvement; stakeholder hierarchy; mutual distrust; preferred stakeholders; and no culture of engagement.

### Tokenistic involvement

Multiple interviewees described their involvement in the government’s policy and budget-making processes as tokenistic (P9, P17, P12). They recounted how they were invited to a consultation event but felt that the policy had either already been substantively developed or that they received the documents for review too late to provide comprehensive feedback. As a result, they felt undervalued in the process and that their voices were not heard, ‘the documents [NHP II and HSSP II] do not reflect the voices of patients’ (P3, CSO).

A CSO interviewee recalled attending two meetings (a consultation and a validation meeting) aimed at discussing the draft strategy. They received the working documents close to the meeting date so there was little time to review them with other network members and prepare a detailed response. They reported giving some feedback during the meetings, but most was ignored. They made a complaint to the MoH, but nothing was done. The members of this network organisation ‘felt that they were consulted as an after-thought just as a requirement to tick the box that the process involved consultation and endorsement of disability organisation or constituencies’ (P17, CSO). Similarly, representatives of Christian Health Association of Malawi (CHAM) facilities were invited to the HSSP II launch event but were not consulted at all during the policy development process. Thus, their involvement was purely to be seen to endorse the policy (P4, government-funded institution).

Three interviews reflected on their experience of the government’s budget consultation meetings (a public consultation event held annually by the Minister of Finance). The events ‘are cosmetic and aimed to coerce stakeholders to rubberstamping an already made budget. Nothing changes in the budget despite people submitting views during the consultations’ (P12, CSO). One NGO expressed the view that ‘meaningful participation is difficult because the Ministry of Finance only announces the budget when they are already at an advanced state of the budget process. At this point it is nearly impossible to make any significant or substantial changes’ (P9, NGO). Another NGO was frustrated by the late point at which they were consulted:

‘nothing can substantially change at this stage of the budget process. [Name of organisation] has not been involved in the budget processes in the past, but we have learnt that if there will be any of our involvement in future, we will need to engage much earlier in the process’ (P9, NGO).

Local government representatives and healthcare providers were equally frustrated with the policy development process (P4, P18, P15, P19). A member of a DHO stated that ‘maybe we were only consulted 20% [of the time] and by the time we were consulted, the process was already at an advanced stage and there was little room to make changes on the issues and content’ (P18, local government). They added that:

‘MoH consultations are more of a window dressing to show the nation that they have consulted with different stakeholders before adopting a national document, but there isn’t much room for external stakeholders (outside the ministry) to influence the process. It’s all about economics […] for me, it’s a waste of time’ (P18, local government).

A local government representative noted that they were only consulted by the government during national health crises which required action at the local level (P19, local government).

There was the perception that Malawi is lagging behind other sub-Saharan African countries in the degree of stakeholder engagement. For example:

‘the situation in Malawi in terms of involvement of persons with disability in policy or budget is different from the situation in Uganda and Kenya, where persons with disability are fully engaged as a priority (not as an after-thought like the case in Malawi). Their input is given attention before finalising the budget’ (P17, CSO).

Even when an NGO took what they felt to be important evidence related to policy to the MoH, there was a lack of interest in what they had to say, ‘[name of organisation] has done some studies on access to health, but their recommendations have not been implemented by government. The recommendations have not been treated with the urgency and importance which it deserves’ (P9, NGO).

Then, where the policy documents did reflect an organisation’s priorities (the affordability, availability and accessibility of health services), there was insufficient detail for the interviewees to be confident that there would be a health improvement (P6, P17). For example, where the specific issue of HIV/AIDS management is mentioned in the NHP II and HSSP II, ‘it is only in passing and without much detail as to how the strategy or plan will manage HIV and AIDS and coordination around it’ (P6, government-funded institution). Likewise, ‘the documents make some reference to disability, but not enough’ (P17, CSO).

### Stakeholder hierarchy

The interviewees’ experiences suggest that not all stakeholders were equal in the exercise of stakeholder engagement. They found a clear hierarchy determined by stakeholder group and knowledge, with donors being the most influential in central government policy-making and CSOs, who had little power, attempting indirect influence via donors.

As donors provide a large proportion of the funding for healthcare in Malawi, they are perceived as having a lot of leverage in policy decision-making, and specifically in driving the development of the NHP II and HSSP II. They were also identified as valuing stakeholder engagement to a greater extent than the government and as making concerted efforts to identify stakeholders and engage them in both the identification of health needs and healthcare implementation (P12, CSO):

‘They [the HSSP II and NHP II were donor driven. They received funding from donors and the donors were influential in the process. They (donors) were active during the consultation and validation meetings. The processes were funded by the donors. The funder influenced the agenda. Follow the money, the money won’t lie’ (P5, NGO).

Thus, the CSOs viewed engagement with donors both as a way to influence their initiatives to meet the needs of the communities they represent and to indirectly influence centralised policy-making. For example, one CSO (P12) is involved in a project with Oxfam because the donor identified them via a stakeholder mapping exercise and invited them to collaborate in further mapping exercises to identify the main needs of citizens and healthcare facilities. The reports from these exercises will be used to evaluate the project’s success and inform the donor’s advocacy agenda (P12).

Having links to donors could limit the opportunities for local level stakeholders to influence policy (although sometimes their desired outcome could still be achieved if a donor independently advocated for it). For example, a CSO had been advocating for HIV self-testing due to the low usage of HIV testing centres, but ‘there was no progress on the government side until PEPFAR took the matter up by setting up future-funding conditions. They said future funding was on condition of progress on self-testing’ (P10, CSO). Another CSO had not been invited to any consultations on the health budget. As they were solely funded by PEPFAR, they believed that the government did not consider them eligible for a say in national budgetary policy-making, ‘[we] are not invited to budget consultations and have not participated in budget reviews. This is mainly because our funding primarily comes from donor agencies such as PEPFAR. We do not receive any funding from government or the national budget’ (P13, CSO). A local government interviewee voiced the same opinion, ‘because they are not targeted for any portion of the national budget, they are not involved in any processes around its development or allocation towards health’ (P16, local government).

A hierarchy of engagement was also observed in government advisory committees. A CSO interviewee reported being part of the technical working groups for the National AIDS Commission in the development of national HIV policy and the HIV and AIDS Act. On the HIV issues, they felt that their ‘issues were taken aboard’ (P13, CSO). However, their influence was still limited as management of the national HIV/AIDs response, and thus most of the decision-making power and influence, remains with the government and donors, who ‘meet regularly to discuss progress in implementation of the HIV and AIDS response, funding mechanisms and alignment with the national policies and strategies’ (P6, government-funded institution).

Despite the considerable leverage of donors over the government, an NGO noted that in health policy development the power to determine the content ultimately remains with the MoH, ‘the Ministry of Health is too bureaucratic and only operates through its technical working groups and related structures. The Principal Secretary sometimes heads the technical working groups, thus maintaining authority and upper-hand in the processes’ (P9, NGO). The government was also perceived as constraining influence from local level stakeholders by holding most of the policy development and consultation events in the MoH buildings in Lilongwe:

‘involvement during the processes for developing the NHP II and HSSP II mostly happened at the MoH headquarters level. There were times when the District Health Office would be involved. Teams from the Ministry headquarters would go to the districts with a questionnaire to ask questions related to policy and strategy. Consultations to finalise the two documents mostly happened at the Ministry headquarters’ (P15, local government).

Hierarchical dynamics were also observed in health decision-making at the district level. Observing the greater value placed on the contributions of those with professional expertise to health decision-making diminished the confidence of CSOs in the processes of engagement:

‘although [name of organisation] members are found all over the country, the secretariat only engages with district authorities minimally due to limited capacity and lack of authority and leverage to influence the District Health Officer who are viewed (by district-level stakeholders) as more senior than the members of [name of organisation]. Most community-based organisation’s leaders do not have the same level of professional expertise as the district health officers (who are doctors) and as such, they feel inferior when engaging with them’ (P10, CSO).

It is worth noting that whilst local level stakeholders want to influence central policy, they are not always open to top-down influence over their own activities. For example, a local government representative reported that:

‘some of the stakeholders are cooperative and listen to directions and advice from the District Health Office in terms of overall district priorities, plans and strategies, but some do not and do their own activities based on their own preferences and priorities […] Most of them [CSOs] do not follow the essential health package and district plans’ (P18, local government).

Likewise, donors exert influence on the government at the central level, including in the extent of stakeholder engagement, but the government may not have much influence on donors at the district policy or implementation level, ‘we are able to collaborate and coordinate with donors at the central level. But at the district level, they are doing their own thing’ (P6, government-funded institution).

### Mutual distrust

There was evidence of mutual distrust between stakeholders which may influence their willingness to engage and be engaged in consultation processes and their effectiveness in them.

The development of the NHP II and HSSP II were considered ‘a MoH thing. It’s also very political’ (P20), with the documents developed to appeal to donors rather than to affect and enforce the changes needed to improve the health and wellbeing of the people of Malawi (P5, P6, P20). The lack of trust between the government and stakeholders may have inhibited meaningful engagement, ‘there is lack of trust between CSOs and government and this affects collaboration’ (P14, CSO).

Concerns were raised about the motives of politicians in policy-making, ‘government decisions suffer political interference from politicians who push government’s decisions towards their interests and preferences’ (P14, CSO). Suspicion of intentions as inhibiting meaningful engagement was also felt to impact government perceptions of CSOs, in particular ‘government officials are less able to collaborate with civil society because they are always suspicious of recommendations of CSOs that they aim to undermine government’ (P14, CSO). The stakeholders thought perhaps the MoH did not involve NGO/CSOs in policy development more because they are perceived as trying to push their own agendas, ‘collaboration among civil society is challenging because stakeholders also want to get a share of the budget of any initiative’ (P12, CSO). This concern may be warranted as, for example, one NGO reported following the policy development and budgeting processes closely to ensure their own project was included in the HSSP II and received funding (P5, NGO).

### Preferred stakeholders

The government was perceived as having a very small group of preferred stakeholders who they engaged repeatedly in the policy development process. This practice benefitted CHAM, the largest non-government provider of healthcare in Malawi, with whom the government routinely consulted on health policies and who would be directly affected by them:

‘when the government is developing policy or strategy documents, they invite CHAM secretariat to the consultation of validation meetings. Prior to attending government consultations, CHAM may call for meetings of its membership for their input e.g. CHAM has previously convened its members to discuss Human Resources for Health policy to seek their input. This is because issues of human resources have been very topical among CHAM members. Many CHAM members were invited to this discussion at a consultative meeting probably because it involved welfare of staff at CHAM member facilities.’ (P4, government-funded institution).

Preferred stakeholders were often identified for public consultation events, such as those held for the HSSP II and annually for the health budget, by including named stakeholders in the open invitation. For example:

‘the budget publicises the budget through a media advert in which they call for interested parties to attend budget consultative meetings held in all districts across the country, and in the advert they mention the stakeholders who they want to attend the consultations. However, the budget consultations are open to anyone who is able to attend’ (P9, NGO).

The practice was perceived as sometimes working well for policies in specific disease areas, particularly when the CSO was involved at all stages of the policy formulation process. For example, one CSO was invited to represent and coordinate all CSOs with an interest in HIV/AIDs in the development of the HIV and AIDs Act and the National Strategic Plan for HIV:

‘the role included coordinating civil society input during consultation, review and implementation processes. They sought input from civil society and represented such voices during consultations’ (P10, CSO).

However, more often, the practice of preferred representation was seen as both directly and indirectly excluding other stakeholders. For closed (invitation only) events those outside health are typically not invited, ‘there is some narrow thinking that health is cross cutting only with nutrition, agriculture and the environment. The health sector only restricts engagement to those working in the health sector when dealing with health matters’ (P14, CSO). Potentially inadvertent exclusions can also arise. In one example, an organisation may not have been invited to an HSSP II consultation meeting due to confusion over the composition of the devolved local government:

‘we were not fully involved perhaps because they assumed [location of local government institution] was represented in the processes. They assumed that we fall under the District Health Office and thought that by including the district health office, we were represented’ (P16, local government).

For open events, by specifying stakeholders who they would like to attend, unnamed stakeholders may feel unwelcome or undervalued in the consultation process, ‘their argument is that it’s an open invitation they put in newspapers so that anyone can attend. I wouldn’t say I went. I attended once but I wasn’t like, we were invited’ (P2, NGO). This NGO representative thought that by holding open invitation events the government could avoid criticism of the representativeness of the stakeholders involved as they would, therefore, be self-selecting. However, to ensure appropriate representation, another interviewee felt strongly that specific stakeholders should be invited to get involved and more comprehensively in the policy development process. They felt this was the only way to ensure inclusion of the voices of patients in the HSSP II as public consultation exercises made a show of stakeholder engagement but did not influence the substance of the policy:

‘the organisation was not involved in any of the processes. They were not invited to any of the consultation or validation conferences. As a result, they even wrote a letter to the Ministry of Health protesting the lack of representation of patients’ groups and voices in the processes leading to the adoption of the two documents’ (P3, CSO).

Other deep concerns about representation were raised. Whilst open events mean that anyone can attend, they do not ensure representation of an appropriately wide range of stakeholders and people from rural communities and vulnerable groups are likely to remain excluded (P3, P12, P14). To elicit a response from a greater number and more varied NGO and CSO stakeholders, using the example of the budget, a CSO recommended that the government ‘circulate a tool with leading questions to seek views on what stakeholders want to see for a budget to be a citizens’ budget’ (P12, CSO). This could overcome any issues of CSOs not having the resources to attend consultation events. For closed events, the central government was perceived as having a preferred CSO partner, the Malawian Health Equity Network (MHEN), which has:

‘regular interface with government for consultations through biannual and ad hoc meetings. They have a good rapport with government. In addition, MHEN Director holds many ongoing confidential bilateral discussions and engagement with government officials to discuss issues as they arise and offer views or advice before decisions are taken’ (P1, CSO).

There was acknowledgement of the suitability (and ease) of using MHEN as the ‘go-to’ CSO for stakeholder engagement: ‘in terms of civil society alliances, MHEN is the most active in convening civil society in the area of health’ (P12, CSO). There was support for its inclusion in a range of MoH committees and, therefore, its ability to influence policy-making across the health sector:

‘MHEN is a member of the government committees on health, including the Health Financing Committee, Community Health Technical Working Group, Human Resources for Health Technical Working Group. Through these ongoing engagement and participation in working groups, MHEN is able to represent its network and bring the voice of the people to the national decision-making processes related to health’ (P1, CSO).

Yet, there was a perception that its involvement meant the exclusion of other CSOs, and that it could not be considered representative of all CSOs:

‘the challenge with Ministry of Health’s engagement with NGO stakeholders is that they assume that MHEN is the representative of all health NGOs, but not all NGOs doing work in the area of health are members of MHEN. The organisation’s view is that MHEN can’t replace grassroots voices in the engagement with the MoH. MHEN does not have capacity to represent all voices, simply impossible’ (P3, CSO).

Further, MHEN was criticised for not adequately representing all the CSOs in its own network (P3, CSO). Instead of all or a representative group of MHEN network members attending policy development activities to represent the CSO and citizens’ voice, the secretariat would attend, ‘the secretariat represents the voices of its member organisations, and through that process they speak on behalf of the people in Malawi’ (P1, CSO). The use of an individual to represent the views of a diverse group of CSOs was viewed as problematic by some organisations (P10 and P3). An alternative approach, proposed to increase CSO influence and representativeness, was for a member of each CSO in a network CSO to attend the consultation event when they wanted to influence central government policy:

‘as a network, not participating in the budget processes is a missed opportunity because it is not only the secretariat that must attend, but the members. If members attended, for example, it could mean more voices on HIV on the budget process. The network has potential to influence the health budget using its leverage of having too many stakeholders in its network. If members attended, there would be a [name of organisation] member at every platform available’ (P10, CSO).

### No culture of engagement

The interviewees believed there to be an absence of an established culture of stakeholder engagement in Malawi, acknowledging that the benefits to stakeholder engagement in policy-making could only be realised via significant changes in thinking and practices by both government and stakeholders: ‘the challenge with Malawian civil society is that they are only reactive in their advocacy. They only react to problems late when a crisis has already occurred, not actively identifying issues and then doing something about it’ (P12, CSO). Examples were given of how individual stakeholders have attempted to overcome perceived barriers to stakeholder engagement to try and influence the government and increase the representation of local level organisations in government health decision-making.

All the interviewees voiced frustration with poor communication from the government around health policy. For example, ‘the major challenge is that government is too bureaucratic and that affects how government is able to communicate with stakeholders and how it provides access to government information. Currently it is difficult’ (P14, CSO). One organisation attributed the absence of a stronger public movement for engagement in policy development to poor government-driven communication. To work around this issue, they had placed members of the MoH on their boards rather than waiting for the MoH to consult them. This provided an opportunity for the MoH to hear about the challenges faced by these facilities and their needs and the needs of the communities to which they provide healthcare (P4, government-funded institution). This organisation has also introduced stakeholder engagement measures into their own decision-making processes ‘to enhance meaningful engagement with its members so that they participate more in decision-making’ (P4, government-funded institution). They now have:

‘several committees through which the secretariat engages with the CHAM members’ facilities. Under that arrangement, CHAM facilities are able to interact between each other, and also get involved in CHAM’s decision-making bodies through input into the committee resolutions which feed into CHAM’s decisions. CHAM also hold regional meetings where it meets and engages with its members at the regional level for information sharing and consultations’ (P4, government-funded institution).

In the face of little perceived effort by central government to engage stakeholders, some CSOs are trying to build bottom-up pressure for stakeholder engagement. For example, MHEN was reported as encouraging additional other stakeholders (as well as their members) to attend consultation events (P20 and P3). There were further examples of CSOs encouraging and modelling stakeholder engagement, such as:

‘[name of organisation] has held meetings to develop standards of care for victims and the last meeting that they had was held in [location] to come up with minimum standards of care for victims of gender-based violence and roles of stakeholders in areas such as police support and healthcare’ (P9, NGO).

These practices were also demonstrated and funded by donors. For example, one CSO referred to a 2 year project funded by Oxfam which aimed to increase the responsiveness of primary healthcare providers to citizens by performing stakeholder analyses to identify health service delivery issues that are of concern to the community. They will then approach the district health office and health service providers ‘to seek corrective action for improvement of services’ (P12, CSO).

Another way of increasing stakeholder engagement was through raising policy literacy, as a lack of understanding about the purpose and process of health policy and budgets was considered a major barrier to influencing health decision-making. It was hoped that building knowledge would lead to advocacy:

‘Although persons with disability have strong voices; they have little knowledge about health and advocacy processes which plays a role in the lack of involvement in health matters. They simply have average knowledge about the relationship between disability and health policy, budgets and broader issues. As such, their advocacy is weak and could be strengthened’ (P17, CSO).

However, ultimately it was felt by the CSOs that the government should be building the culture of engagement by providing opportunities for meaningful engagement (P3 and P12). This is despite the development of a patients’ welfare charter. As one interviewee ruefully stated, ‘the idea of a patients’ welfare association is to give people their rights to be involved in decisions about their health. The Ministry of Health previously developed a patients’ welfare charter, but it was never implemented’ (P3, CSO). It was hoped that the implementation of the charter might boost citizen engagement in health advocacy, which is especially important in a distributed healthcare system where the needs of citizens in rural villages vary from those in urban settings, ‘the importance of protecting patients’ rights is even more critical in the villages where patients view public health services as a favour from government, as opposed to viewing such services as obligations enshrined in the constitution’ (P3, CSO). Accordingly, citizens continue to feel voiceless in health policy decision-making.

## Discussion

We have identified that local level government and civic and social organisations in Malawi want to be meaningfully involved in central government health policy processes and believe that they should be. Their goal is to ensure that the needs of the communities they represent are better met. When directly invited to participate in consultation exercises, stakeholders generally did (including for the HSSP II – none were invited to consultations on the NHP II); but they frequently felt that their engagement in consultation events was tokenistic and that the government typically engaged a small number of preferred stakeholders, resulting in a lack of representation for others.

The Malawian MoH consulted a range of stakeholders via technical working groups, consultation events and visits to institutions, departments and programmes in the development of the NHP II and HSSP II. Those consulted included CSOs, NGOs and local government, namely the DHOs. Walsh et al.’s analysis of the policy development process assessed it as ‘complex and inclusive’, with DHOs involved in most and CSOs in all stages of the process [19]. However, the individuals or names of the organisations engaged, the method and extent to which each was involved, and the impact of their input was not reported. Our study highlights a discrepancy in the reported and perceived inclusivity of the policy development process from the perspective of local level stakeholders. This discrepancy and the challenges to stakeholder engagement that we have identified are not unique to Malawi or even to developing countries, and not only to health policy.

We highlight the transferability of our findings by relating them to the wider discourse on stakeholder engagement in policy development processes in LMICs, particularly in other sub-Saharan African countries. Our discussion focuses on inclusive and meaningful engagement and how it could be facilitated in practice.

First, we acknowledge some methodological issues that may affect the transferability and repeatability of our study and findings. A range of stakeholders from local level organisations were recruited to the wider study, although there were more representatives from CSOs than the other organisation types largely due to the exclusion of transcripts from three local government representatives. As a result, and because we reported all stakeholder perceptions regardless of the number of individuals to comment on each issue, greater space was given to issues identified by CSOs than local government representatives. The sample was relatively small and unbalanced by organisation type, yet the inclusion of two or more interviewees within each organisation type gave some indication of potentially common concerns within organisation types.

The need to protect the identities of the interviewees limits the transferability of the findings [20]. The semi-structured interview approach increased the breadth of the data collected but reduced the repeatability of the study. The interviews were recorded via field notes and transcripts which were not participant-verified. We followed guidelines for making field notes although the mitigation of the risk of interviewer subjectivity in the recording and transcription of the field notes was impossible [20]. The interviewer bias was limited as they had not previously worked in the health sector and had lived outside Malawi for some time. Instead, their Malawian nationality and health sector ‘outsider’ status may have engendered more open disclosure from the interviewees.

### Inclusive engagement

National health policies and accompanying strategic frameworks are used to give direction and coherence to national efforts to improve health [7]. In developing countries which may have fragmented healthcare systems, like Malawi, health policy coordination is necessary to increase accountability and governance of the healthcare system and to align the activities and budgeting of internal (e.g. the government) and external (e.g. donors) funders [7, 17]. It is widely understood that the purpose of these policies is to address the wider public health agenda, going beyond healthcare delivery. This necessitates consultation with multiple stakeholders to identify the needs of the population, including marginalised groups and considering stakeholders adjunct to health (e.g. housing, environment, education, nutrition), reflecting them in the policy [7, 21].

Yet, it is common in policy consultations for some stakeholders to have greater power, knowledge and resources to affect the policy content, particularly in LMICs with highly centralised policy-making and where a significant proportion of the national budget often comes from other national governments or donors [1, 2, 22, 23]. The donor dependency of the Malawian health sector means that through donor-government partnerships international development agencies can ‘lead from behind’ to ensure national policies and initiatives align with their own preferences [24]. It was suggested in our study and in literature on high and low income countries that the extent of the influence possible in centralised policy-making is relative to the financial contribution made by the stakeholder [25–27]. Limited government funding may also explain limited local level engagement and the use of preferred partners, perhaps inviting partners with the independent means to attend consultation events held in the MoH headquarters in Lilongwe [25].

Our interviewees raised concerns about the representativeness of one network organisation (MHEN) repeatedly invited by the MoH to represent CSOs and marginalised groups when their membership does not include every CSO in Malawi. For example, people with disabilities did not feel represented. The seeming monopoly of representation by a few individuals and CSOs who typically attend stakeholder consultation events has been noted in another study from Malawi and in the literature on global policy making [28, 29]. This is likely to reflect a hierarchy in the value placed on the contributions of different stakeholders by the government, and has also been noted in research from other African countries, such Zambia and Uganda [25, 30]. A hierarchy of engagement often prevents the most relevant stakeholders being approached for participation (e.g. women, rural and marginalised groups) and gives less weight to the contributions of stakeholders perceived as lower in the hierarchy [25]. Their contributions are perceived as less accurate or useful than other forms of evidence used in the decision-making process [10, 31].

### Meaningful engagement

We understand meaningful engagement as opportunities for communication, consultation and participation that diverse stakeholders consider accessible, timely, and through which they are as able to exert influence. Our interviewees reported instances of communication, consultation, and participation but there was no to-way dialogue with the MoH about the needs of their communities and the policy content. They were seen to be consulted and positioned to endorse an already finalised policy rather than influencing its content. Thus, there was engagement, but it was not meaningful, leading to disappointment with the final policy and its implementation which is unlikely to address existing health inequalities [5, 31].

Donors were generally viewed as making greater efforts than the MoH to consult local level organisations to inform their work, and many of the calls for inclusive and meaningful stakeholder engagement have come from donors such as the WHO and the United Nations who were partners in the development of the HSSP II [9, 32]. Walsh et al. [19] found that to ensure the continued involvement of donors, the MoH may have given up ownership of the (HSSP II) policy development process to them, a practice which is not uncommon in sub-Saharan African countries [33]. However, despite the significant influence and likely leadership of donors in the NHP II and HSSP II process, the local level stakeholders still perceived their engagement as tokenistic, with rushed consultation which prevented deeper engagement, and limited ways of contributing such as alternatives to attending a public consultation event in person [10, 22, 31, 32]. These issues might have been mitigated if the donors’ own guidance on stakeholder engagement had been implemented. For example, WHO advise that:

‘national health policies, strategies, and plans are more likely to get implemented effectively if their development and negotiation is inclusive of all stakeholders in and beyond the health sector. This means engaging all actors by means of a broad consultation in meaningful policy dialogue to build consensus on the current situation and on the values, goals and overall policy directions that will guide health policy’ [9].

The effective engagement of local level organisations, including citizens and communities, in policy-making can reinforce democratic ideals and institutions, give the public a sense of ownership of regulation and open up a channel of two way communication between the public and health decision-makers in the government [34]. There have been efforts to engage communities and citizens in local policy-making to ensure the policies meet their needs, although they have been hampered by the retention of power over local decision-making by the centralised government, due to the incomplete implementation of the policy decentralising powers to the district level [17, 34–36]. As observed in our study and in health priority setting in Uganda, the result may be an obscuring of local government voices in deference to centralised government decision-makers in MoH policy-making [30]. Although DHO representatives were included in most stages of the policy development [19], we found that local government representatives (including some DHOs) felt under-engaged in the process and powerless to influence the content. Further, within the context of decentralisation uncertainty, if community engagement is more usually performed and engaged with at the local government level it may explain, but not excuse, the limited engagement of communities and citizens in the national policy process [34].

To our knowledge there has been no significant strengthening of stakeholder engagement in the centralised policy development process since the publication of the NHP and HSSP II in 2017. The only publicly available government-sanctioned stakeholder engagement plan we have found is for the Malawi Covid-19 Emergency Response and Health Systems Preparedness Project and was developed by the World Bank together with the government [37]. Here again, it seems that a donor rather than the government is asserting the role of stakeholder engagement in health decision-making in Malawi [38].

### Implications for policy and practice

For stakeholder engagement in the development of health policy in Malawi to be more inclusive and meaningful, those with the responsibility and power for drafting and finalising health policy must be inclusive in their approach and responsive to all stakeholder contributions [4, 32]. In developing the NHP II, the government of Malawi used a narrow interpretation of health stakeholders as only those directly involved in the functioning of the health system, such as DHOs, central hospitals, health donors and CSOs. However, the policy is closely aligned with Sustainable Development Goal Three to ensure healthy lives and wellbeing for all at all ages [15]. We suggest, therefore, that all people of all ages are stakeholders in health policy and should have the opportunity to engage in the policy development process. It is argued that only through the engagement of underrepresented groups in policy-making can the perpetuation of health inequalities be avoided [39]. The principle of ‘nothing about us, without us’ should be applied, where the engagement of those who suffer the most from inequalities is prioritised in the policy design process [40].

Two way communication between the government and stakeholders, inclusive of marginalised groups, throughout the policy development process is essential for building trust and social accountability [29, 41–43]. This should include feedback mechanisms at the implementation stage so stakeholders, especially CSOs and healthcare facilities can identify where the policy is not meeting the population needs [2]. This mechanism would give the local level stakeholders more power and impact in the policy development process and implementation. This, and transparency about the aims and expectations of each stakeholder can increase confidence in the process of working together to achieve the best possible health outcomes rather than each stakeholder trying to satisfy their own agenda [21, 22], concerns which were raised by our interviewees.

Low policy literacy in the population is frequently cited as a barrier to the inclusion of local level stakeholders in policy development particularly in LMICs [10], including in our study. Health policy decision-making is often a complex process involving technical information, so an educational component relating to the content as well as the process may be necessary for stakeholders without a clinical background [44]. To facilitate the engagement of stakeholders with different levels of education, policy literacy and in marginalised groups, including socioeconomically disadvantaged and rural communities, comprehensive stakeholder engagement will always require the deployment of multiple techniques. In this, guidance and resources developed by NGOs, donors and academics for use in LMICs could prove valuable [3, 45], such as toolkits to enable effective engagement with stakeholders and the use of engagement techniques such as Bwalo forums and CARE scorecards which have been used successfully in Malawi and Kenya [10, 21, 46–51]. There are also lessons on how to increase citizen engagement in policy-making from the Mwananchi project which sought to enhance social accountability to deliver pro-poor policy changes in six African countries, including Malawi, by changing citizen-government relations [52]. Frameworks and guidance on stakeholder engagement in general and on policy-making specifically are available [2, 7, 10, 48]. The majority focus on maximising community participation via stakeholder analysis (e.g. 1) or direct consultation (e.g. 23), but typically overlook the potential challenges to participation experienced by other key stakeholders, such as local government [29].

Inclusive engagement approaches must combine with political advocacy activities if local level stakeholders are to achieve sustainable and meaningful engagement in national decision-making [52–54]. Despite the democratisation process taking place in Malawi, the enduring legacy of the strong presidency remains a huge impediment to subjecting the policy-making processes to the influence of a diverse range of stakeholders so that they become as participatory, transparent, and accountable as possible [11]. There are signs of a burgeoning people’s power movement in Malawi which may result in louder calls for more meaningful, particularly citizen, stakeholder engagement [55]; although the greater investment of power in civil society organisations and their continued cooperation with donors may be necessary for sufficient pressure to be placed on the government [56, 57].

Further research is warranted to understand the behaviour, dynamics, interrelations, agendas and influence occurring between citizens, communities and the different levels of government (ministerial, regulatory bodies, local government, service providers, CSOs, donors). We are also interested in if and how the extent of stakeholder engagement varies depending on the specific policy and what lessons might be transferable between sectors and disease areas to enhance stakeholder engagement in health policy-making.

## Conclusion

We have identified high levels of dissatisfaction in local level stakeholders with issues of meaningful and inequitable engagement of multiple stakeholder groups throughout the health policy process. These stakeholders felt that due to a lack of top-down and bottom-up pressure for more comprehensive stakeholder engagement, there was tokenistic consultation with local level stakeholders. They had no power to influence health policy-making to better meet the needs of the communities they represent. Local level stakeholders can be empowered to advocate for and participate in both invited and open MoH stakeholder engagement exercises, and policy literacy increased but greater top-down efforts to engage stakeholders via diverse and inclusive methods are also required. These themes are not isolated to Malawi but are also identified in other LMICs. Existing resources from donors and academia can be used to support inclusive engagement including boundary and expectation setting for both the MoH and health stakeholders to build mutual trust and cooperation between all interested parties.

## Data Availability

The datasets generated and analysed during the current study are not publicly available as analysis is ongoing for additional publications but are available from the corresponding author on reasonable request.

## Abbreviations

CHAM: Christian Health Association of Malawi
CSO: Civil Society Organisation
LMICS: Low and middle income countries
MHEN: Malawi Health Equity Network
MoH: Ministry of Health
NAC: National AIDS Commission
NGO: Non-government organisation
NHP: National Health Policy
HIV: Human immunodeficiency virus
HSSP: Health Sector Strategic Plan
